# Brazilian Society of Rheumatology’s fibromyalgia treatment guidelines – part I: monitoring and non-pharmacological management

**DOI:** 10.1186/s42358-025-00507-x

**Published:** 2026-01-16

**Authors:** Roberto Ezequiel Heymann, Marcelo Cruz Rezende, Alessandra de Sousa Braz, Aline Ranzolin, Ana Paula Monteiro Gomides Reis, Andrea Pimentel Fonseca Golmia, Anna Beatriz Assad Maia, Denison Santos Silva, Eduardo dos Santos Paiva, Fernando Augusto Chiuchetta, Gabriela Tannus Branco de Araújo, Izabela Guimarães, José Eduardo Martinez, José Roberto Provenza, Juliana Maria de Freitas Trindade Costa, Marcos Paulo Veloso Correia, Marco Antonio Gonçalves Pontes Filho, Marco Aurelio Goldenfum, Marcos Aurelio de Freitas Machado, Marcos Renato de Assis, Melissa Mariti Fraga, Nilton Salles Rosa Neto, Rafael Navarrete Fernandez

**Affiliations:** 1 Hospital Israelita Albert Einstein, São Paulo, SP Brazil; 2 Pain specialist certified by the Brazilian Medical Association, Campo Grande, MS Brazil; 3 Hospital Universitário Lauro Wanderley, Universidade Federal da Paraíba, João Pessoa, PB Brazil; 4 Hospital das Clínicas de Pernambuco, Recife, PE Brazil; 5 Centro Universitário de Brasília, Brasília, DF Brazil; 6 University Hospital of Brasília, Brasilia, DF Brazil; 7 Universidade Tiradentes, Aracaju, Sergipe Brazil; 8 Universidade Federal do Paraná, Curitiba, PR Brazil; 9 Hospital da XV, Curitiba, PR Brazil; 10 Axia Bio MSc, São Paulo, SP Brazil; 11 Pain specialist certified by the Brazilian Medical Association, Florianopolis, SC Brazil; 12 Pontifícia Universidade Católica de São Paulo, São Paulo, SP Brazil; 13 Pontifícia Universidade Católica de Campinas, Campinas, SP Brazil; 14 Hospital de Aeronáutica de Recife, Recife, PE Brazil; 15 Universidade Federal do Rio de Janeiro, Rio de Janeiro, RJ Brazil; 16 Pain specialist certified by the Brazilian Medical Association, São Paulo, SP Brazil; 17 Hospital São Lucas, Pontifícia Universidade Católica do Rio Grande do Sul, Porto Alegre, RS Brazil; 18 Member of the International Association for the Study of Pain, Ribeirão Preto, SP Brazil; 19 Faculdade de Medicina de Assis, Assis, São Paulo Brazil; 20 Universidade Federal de São Paulo, São Paulo, SP Brazil; 21 Universidade Santo Amaro, São Paulo, SP Brazil; 22 Pontifícia Universidade Católica de Goiás, Goiânia, GO Brazil; 23 Member of the Pain Committee of the Brazilian Society of Rheumatology, São Paulo, Brazil

**Keywords:** Fibromyalgia, Chronic pain, Non-pharmacological treatment, Central sensitization, Nociplastic pain, Patient monitoring, Exercise therapy, Multidisciplinary approach, Cognitive behavioral therapy, Aerobic exercise, Strength training, neuromodulation, Pain assessment tools, guidelines

## Abstract

Fibromyalgia is one of the most prevalent rheumatologic conditions, affecting millions of individuals worldwide. It is characterized by widespread chronic pain accompanied by symptoms such as fatigue, sleep disturbances, and cognitive impairments. In Brazil, fibromyalgia is the second most common rheumatologic disorder, with a reported prevalence ranging from 2.5% to 5.5%, leading to a substantial economic burden due to high direct and indirect healthcare costs. The pathophysiology of fibromyalgia involves alterations in central pain processing, often in conjunction with comorbidities such as depression, anxiety, and irritable bowel syndrome, which exacerbate symptom severity and negatively impact patients’ quality of life. Effective management requires a structured, interdisciplinary approach that emphasizes treatment adherence and patient education. This guidelines from the Brazilian Society of Rheumatology (SBR) provide an updated review of the 2010 recommendations, incorporating recent evidence to refine both pharmacological and non-pharmacological therapeutic strategies, and are presented in two separate articles, covering aspects of patient monitoring, non-pharmacological interventions, and pharmacological treatments. This approach underscores commitment to comprehensive, evidence-based, and patient-centered care. This first article focuses on the evaluation of monitoring strategies and non-pharmacological interventions for managing fibromyalgia.

Fibromyalgia is one of the most prevalent rheumatologic conditions, affecting millions of individuals worldwide. It is characterized by widespread chronic pain accompanied by symptoms such as fatigue, sleep disturbances, and cognitive impairments. In Brazil, fibromyalgia is the second most common rheumatologic disorder, with a reported prevalence ranging from 2.5% to 5.5%, leading to a substantial economic burden due to high direct and indirect healthcare costs.

The pathophysiology of fibromyalgia involves alterations in central pain processing, often in conjunction with comorbidities such as depression, anxiety, and irritable bowel syndrome, which exacerbate symptom severity and negatively impact patients’ quality of life. Effective management requires a structured, interdisciplinary approach that emphasizes treatment adherence and patient education.

This guidelines from the Brazilian Society of Rheumatology (SBR) provide an updated review of the 2010 recommendations, incorporating recent evidence to refine both pharmacological and non-pharmacological therapeutic strategies, and are presented in two separate articles, covering aspects of patient monitoring, non-pharmacological interventions, and pharmacological treatments. This approach underscores commitment to comprehensive, evidence-based, and patient-centered care.

This first article focuses on the evaluation of monitoring strategies and non-pharmacological interventions for managing fibromyalgia.

## Introduction

Fibromyalgia (FM) is one of the most prevalent rheumatologic diseases, affecting millions of individuals worldwide. It is primarily characterized by chronic and widespread musculoskeletal pain, often accompanied by other debilitating symptoms such as persistent fatigue, sleep disturbances, morning stiffness, paresthesias in the extremities, sensations of edema, and impairments in cognition and functionality [1, 2]. In Brazil, FM is the second most common rheumatologic condition, surpassed only by osteoarthritis, with an estimated prevalence ranging between 2.5% and 5.5% [3–5]. Although the etiology and pathogenic mechanisms of FM are not yet fully understood, alterations in central pain processing are widely recognized as playing a pivotal role [1]. These dysfunctions are frequently associated with comorbidities such as depression, anxiety, chronic fatigue syndrome, myofascial pain syndrome, irritable bowel syndrome, and nonspecific urethral syndrome, all of which contribute to symptom exacerbation and further impair patients’ quality of life [1, 2] The systemic impact of FM leads to increased healthcare utilization and extensive use of analgesic treatments, generating substantially higher direct and indirect costs including consultations, diagnostic tests, medications, absenteeism, reduced productivity, and early retirement. [6, 7].

Structured treatment monitoring is essential for tracking symptom progression, adjusting therapeutic interventions, preventing adverse effects, and enhancing patient adherence. Additionally, it facilitates educational support and improves disease management. A interdisciplinary approach, involving various healthcare professionals, is crucial for comprehensive care.

Over the past three decades, significant advances have improved the understanding of FM mechanisms and therapeutic strategies, particularly non-pharmacological interventions. The 2010 Brazilian Society of Rheumatology (SBR) guidelines emphasized both pharmacological and non-pharmacological treatments, with subsequent studies reinforcing their efficacy [8]. These updated recommendations revise and expand the 2010 guidelines, integrating the latest advancements to enhance patient outcomes. The SBR reaffirms its commitment to evidence-based, comprehensive care, providing a practical tool for healthcare professionals to optimize FM management.

For clarity and organization, these guidelines are divided into two articles. The first focuses on the reassessment of patient monitoring methods and non-pharmacological treatment strategies, emphasizing evidence-based interventions for effective management. The second article provides an updated review of pharmacological approaches, incorporating the most recent advances in the field.

## Materials and methods

The methodology adopted in this guideline was based on the BASCE System (an acronym for Search, Analyze, Select, Classify, and Elaborate) [9], an organizational method developed by the consulting firm Axia.Bio. The primary objective of this system is to minimize deviations and biases in the results by employing criteria grounded in consolidated scientific literature. It provides a systematic approach to consensus-building, addressing issues relevant to the local context. In this study, the process focused on evaluating systematic reviews and meta-analyses of the literature and on the voting of SBR experts to answer the formulated questions. The process followed the key steps outlined below:

1. **Broad and systematic search in the medical literature:** Meta-analyses and systematic reviews from the last 10 years related to FM were identified.
2. **Structured evaluation of studies:** A panel of local experts (Group I) selected the most relevant materials for the proposed questions, using a scoring system for prioritization.
3. **Review and group voting:** A second group of experts (Group II) voted on the recommendations based on the findings and their applicability in Brazil, ensuring consensus.

### Process stages

#### First stage: preparation of questions and bibliographic research

SBR specialists defined 31 key questions focusing on the therapeutic monitoring and treatment of fibromyalgia. Based on these questions, a bibliographic search was conducted in databases such as PubMed, LILACS, and the Cochrane Library, employing specific search strategies.

The search strategy in PubMed and LILACS was as follows: (“fibromyalgia” [MeSH Terms] OR “fibromyalgia”[All Fields]) AND systematic[sb] AND (“1”[PDAT] : “2008/06/13”[PDAT]) AND (English[lang] OR Spanish[lang] OR Portuguese[lang]), yielding 238 publications. In the Cochrane Library, the term “fibromyalgia” identified 51 publications.

After excluding duplicate references, the relevant publications were forwarded to six experts (Group I), who evaluated and voted on the relevance of the studies in addressing the proposed questions. Only studies achieving an agreement level of 70% or higher proceeded to the next stages. Experts also considered the degree of recommendation and applicability in Brazil when selecting references.

#### Second stage: voting on the recommendations

In this phase, fibromyalgia specialists convened to vote on whether to agree or disagree with the statements derived from the selected studies. Group II comprised the members of Group I along with additional specialists appointed by the SBR Pain Commission, totaling 22 SBR specialists.

Voting was conducted electronically and anonymously using the Mentimeter (https://www.mentimeter.com) platform. Recommendations were formulated based on statements that received at least 70% agreement (YES or NO). For statements that did not reach consensus in the first round, a debate was held between supporting and opposing experts, followed by a second vote. Questions failing to meet the required percentage in the second round were classified as lacking consensus and were excluded from the final recommendations.

All 31 proposed questions underwent voting, and all achieved consensus among the experts. Consequently, the practices recommended in this guidelines have at least 70% agreement among participating experts and are supported by bibliographic references. The absence of a recommendation for a specific treatment does not imply its inefficacy or prohibition. This consensus exclusively endorses treatments substantiated by robust scientific evidence.

Consensus documentation, including voting records and results, was archived through the Mentimeter platform, ensuring transparency and traceability.

This first article focuses on the monitoring of FM patients and non-pharmacological therapeutic approaches, covering the first 18 of the 31 analyzed questions.

## Recommendations

### What standardized assessment instruments are available to evaluate the impact of FM and the efficacy of therapeutic interventions in affected patients?

CONSENSUS: The utilization of standardized questionnaires for the assessment of FM patients is recommended due to their essential psychometric properties, with no specific preference for any particular instrument.

A wide range of questionnaires has been specifically developed for the assessment of FM patients, many of which demonstrate good to excellent psychometric properties. Although these instruments exhibit heterogeneous structural characteristics, they provide robust and reliable psychometric evaluations of patients [10].

In Brazil, the *Revised Fibromyalgia Impact Questionnaire (*FIQR) and the *Fibromyalgia Survey Questionnaire* (FSQ) are recommended, as they have been validated for the Portuguese language.

The FSQ evaluates the primary symptoms of FM, including the intensity of somatic symptoms and the distribution of pain sites [11]. It comprises the *Widespread Pain Index* (WPI) and the *Somatic Symptom Severity Score* (SSS), both integral to the fibromyalgia diagnostic criteria established by the American College of Rheumatology (ACR) in 2010 and subsequently modified in 2011 and 2016 [11, 12]. The FSQ’s utility lies in its self-administration format, facilitating data collection in research as well as in clinical environments with limited time and resources. Studies have demonstrated that the FSQ possesses strong internal consistency, along with convergent and discriminant validity, making it a reliable assessment tool [11, 12].

The FIQR is a revised version of the *Fibromyalgia Impact Questionnaire* (FIQ), designed to improve the balance across functional, overall impact, and symptom domains. It includes additional questions regarding memory, sensitivity, and balance [13]. The FIQR is widely employed to evaluate disease severity and functional capacity in FM patients and has been validated across multiple populations, including in Brazil [13, 14].

### What should be the target symptoms of FM treatment?

CONSENSUS: Generalized pain, fatigue, and sleep disturbances are primary target symptoms in the treatment of FM. However, it is crucial to consider additional factors such as hypersensitivity to palpation at multiple anatomical sites, cognitive impairments, mood disturbances, and obesity when formulating an individualized treatment plan.

In 2009, the Outcome Measures in Rheumatology (OMERACT) established the mandatory domains for clinical trials in FM based on the 1990 ACR criteria. These domains include pain, fatigue, sleep disturbances, multidimensional functional limitation, hypersensitivity to palpation (tenderness), and global assessment. Additionally, optional domains for specific clinical trials encompass depressive and cognitive disorders, while anxiety and rigidity were considered domains of scientific interest [15].

A 2022 systematic review concluded that among these domains and their subdomains, the most frequently assessed in FM trials since 2015 (including studies utilizing the 2010, 2011, and 2016 ACR criteria) were: pain (98%), depression (98%), fatigue (96%), anxiety (95%), physical limitation (95%), global health (88%), social participation (86%), sleep disturbances (85%), and rigidity (82%). Cognitive alterations and hypersensitivity to palpation were evaluated in 29% and 17% of trials, respectively [16].

Pain (both in terms of intensity and frequency), fatigue, sleep disturbances, and mood disorders appear to be independent risk factors for the onset or exacerbation of generalized chronic pain. Moreover, chronic pain can exacerbate these factors (fatigue, sleep disturbances, and mood disorders), creating self-perpetuating cycles with significant therapeutic implications. [17]. Emotional state and cognitive perception directly influence pain progression, whereas chronic pain contributes to the development of depressive-anxiety symptoms and cognitive dysfunction [17, 18]. Population-based studies indicate that sleep disturbances are the strongest predictor for the development of generalized pain over 10- to 18-year periods, serving as a more reliable indicator for pain occurrence than the reverse relationship [18–21]. Additionally, fatigue has been identified as a more significant predictor for generalized pain over a five-year period than depression or anxiety [22] and is correlated with CS regardless of pain presence [23, 24].

In 2019, the North American public–private partnership ACTTION–American Pain Society Pain Taxonomy (AAPT) proposed a multidimensional diagnostic framework for fibromyalgia (FM), aiming to complement and refine existing classification criteria such as the 2016 ACR criteria.The AAPT framework defines FM as a chronic primary pain condition characterized by multiregional pain (a mandatory criterion) associated with fatigue and sleep disturbances, but it also emphasizes a five-domain evaluation that includes core diagnostic features, common features, comorbidities, neurobiological mechanisms, and consequences of the condition (e.g., functional impact and family history).This multidimensional model highlights the complex biopsychosocial nature of FM, although further empirical validation is still needed before its broad clinical adoption [25].

It is essential to recognize that, beyond patient-reported symptoms, additional clinical considerations must be integrated into FM management. For instance, functional limitation, including physical impairment, has gained increasing attention, with studies suggesting that improvements in functionality can occur independently of and even precede reductions in pain intensity [26].

### What tools can be used to identify central sensitization in other rheumatic diseases?

The scale derived from the 2016 diagnostic criteria, known as the FM Survey Questionnaire (FSQ), and the Central Sensitization Inventory (CSI) are indicated to identify nociplastic pain and central sensitization in other rheumatic diseases.

Both the Fibromyalgia Severity Questionnaire (FSQ), derived from the ACR2016 criteria, and the Central Sensitization Inventory (CSI), developed from studies on central sensitization and associated chronic pain conditions, have been validated for Brazilian Portuguese. These instruments are valuable for assessing the nociplastic component and generalized central sensitization in both rheumatic and non-rheumatic diseases [27–31].

The CSI is a reliable and practical tool for identifying pain associated with central sensitization (CS) in patients with rheumatic diseases such as rheumatoid arthritis (RA), spondyloarthritis (SpA), osteoarthritis (OA), and fibromyalgia (FM). Evidence indicates that CS is present in a substantial proportion of these patients, often contributing to pain disproportionate to peripheral inflammation.The CSI not only aids in identifying CS-related pain but also serves as a prognostic indicator and a potential guide for precision pain management in rheumatology [30, 31].

The FSQ is a self-report instrument proposed as a low-cost alternative for central sensitization assessment. Research has demonstrated that FSQ scores correlate with Quantitative Sensory Testing (QST) measures in RA patients, although the strength of this association is relatively weak [32].

### Should FM be considered a disease or syndrome?

Consensus: Syndrome is the best term to define FM.

FM is characterized by a constellation of symptoms, with generalized pain as the primary clinical manifestation. Additional symptoms, such as fatigue, sleep disturbances, cognitive dysfunction, and mood alterations, often coexist, adding to the heterogeneity and diagnostic complexity. Despite extensive research, its etiology and pathophysiology remain incompletely understood.

Current models propose that FM results from the interplay of central and peripheral mechanisms. The “top-down” hypothesis emphasizes central nervous system dysregulation leading to amplified pain perception, whereas the “bottom-up” hypothesis focuses on persistent peripheral nociceptive input that contributes to central sensitization. These mechanisms are now regarded as complementary, underscoring the multidimensional nature of FM pathogenesis.

Historically, FM has also been classified as “primary,” when occurring independently, or “secondary,” when associated with other medical conditions such as inflammatory or chronic pain disorders. Although this distinction declined in use, it is regaining relevance in clinical and research contexts.

By definition, a disease implies specific and identifiable causes, whereas a syndrome encompasses multiple possible etiologies. Accordingly, given its multifactorial and heterogeneous nature, the term “syndrome” remains the most appropriate designation for FM. [33–36].

### What is the best translation for the English term “fibromyalginess”?

Consensus: No consensus was reached regarding the optimal translation of the term “fibromyalginess” for use in Brazilian rheumatology.

“Fibromyalginess” represents a spectrum of symptom expressions in fibromyalgia, encompassing pain, fatigue, sleep disturbances, and cognitive impairments. It reflects the continuum along which patients may vary in the intensity and combination of these symptoms, rather than a dichotomous presence or absence of disease. This spectrum-based concept enables a more nuanced understanding of fibromyalgia, supporting personalized assessment and treatment strategies according to individual symptom severity [37, 38].

In a survey conducted within the group, participants assigned the following meanings to the term with corresponding percentages: “Fibromyalginity” received 25% of the votes, the “Fibromyalgia Severity Scale” obtained 15%, the “Fibromyalgia Activity Scale” reached 45%, and the “Somatic Stress Index” accounted for 15% of the votes.

### Is patient and family education an effective intervention in the management of FM?

CONSENSUS: Patient education is considered a fundamental component of the comprehensive treatment plan for FM.

In view of the scarcity of new therapeutic options for FM in recent years, the implementation of health education tools becomes even more relevant, especially to encourage self-care in patients with FM [39].

Studies show that health education has important benefits in reducing emotional symptoms, such as anxiety and depression, as well as clinical symptoms, including fatigue, morning stiffness, and pain intensity. This approach also promotes improvements in overall functional status, encompassing physical function and the ability to cope with fatigue and stiffness [40]. In interdisciplinary health education programs, topics such as information about FM, body practices, physical activities, and pharmacological approaches are often addressed [41].

Psychoeducational programs have been shown to be viable and effective for the control of physical and emotional symptoms, especially in relieving pain and depression [39]. The main goal of patient education is to empower them to manage their own health needs by promoting self-management and facilitating the return to desired daily activities [42, 43].

Education facilitates multidisciplinary integration, with each healthcare professional playing a role in the effective management of FM. Strategies implemented in multidisciplinary groups are essential for addressing the various aspects of this syndrome [41].

### Are diet and nutritional supplements effective in treating FM?

CONSENSUS: A balanced diet is recommended for patients with FM; however, there is no conclusive evidence regarding the optimal dietary strategy. Currently, no definitive evidence supports the efficacy of specific diets or nutritional supplements in the treatment of FM. For patients with obesity, weight reduction is recommended as a component of the comprehensive treatment approach.

Nutritional guidance is an integral component of FM management; however, similar to other interventions, it is not effective as a standalone treatment [44, 45].

Nutritional interventions, including olive oil consumption, ancient grain diets, low-calorie diets, low-FODMAP (fermentable oligosaccharides, disaccharides, monosaccharides, and polyols) diets, gluten-free diets, monosodium glutamate- and aspartame-free diets, vegetarian diets, and the Mediterranean diet, have demonstrated efficacy in alleviating FM symptoms [46–49]. Additionally, evidence suggests that a high-protein diet may increase the pain threshold in FM patients [44].

Although the aforementioned dietary interventions and supplements may contribute to pain reduction in FM, their impact on overall quality of life remains unproven. Reported adverse effects range from mild to severe, with gastrointestinal symptoms being the most frequently observed [45].

Systematic reviews indicate that weight loss, alongside addressing the neuropsychological component of FM, should be considered as part of the treatment approach. Dietary modifications are regarded as a promising complementary strategy for FM management. However, no single dietary approach has been universally recommended [44–49], and the current evidence is insufficient to conclusively support the use of any specific dietary supplement for FM treatment. Further well-designed and rigorously conducted studies are necessary to validate these findings and establish clear guidelines for the use of nutritional supplements in FM management [45].

### Is there evidence supporting the superiority of treatment by a interdisciplinary team compared to medical treatment alone?

CONSENSUS: Interdisciplinary treatment is recommended because it provides improvements in the quality of life of patients with FM.

Interdisciplinary treatment involves the collaboration of several health professionals to offer a more comprehensive and integrated care to the patient. The team may include doctors, nurses, physical therapists, physical education professionals, psychologists, nutritionists, occupational therapists, and other specialists. [41]

Interdisciplinary groups show greater efficacy in several aspects of FM treatment, especially in quality of life and pain relief, when compared to strategies in which each professional acts in isolation [41].

The implementation of protocols incorporating Interdisciplinary discussions and the establishment of quality-of-care indicators have been shown to enhance outcomes in patients with FM, particularly in terms of quality of life [50].

Interdisciplinary health education programs that provide information on FM, body practices, physical activities, and pharmacological approaches have demonstrated efficacy in improving pain and health-related quality of life (HRQoL) in patients with FM [41, 50].

### Which aerobic exercise modalities have demonstrated effectiveness in the management of FM?

CONSENSUS: Aerobic exercises, including walking, running, dancing, cycling, and water aerobics, have been demonstrated to improve the quality of life in patients with FM. Currently, no evidence supports the superiority of one modality over another; therefore, all forms of aerobic exercise should be considered as viable therapeutic options.

A systematic review of 12 studies on FM demonstrated that moderate- to high-intensity aerobic exercise, performed twice per week, reduces autonomic dysfunction and increases heart rate variability [51].

Another systematic review of 12 randomized controlled trials found that both aerobic and aquatic exercises significantly improve the functional capacity of patients with FM, with large and moderate effect sizes, respectively. It is recommended that patients engage in moderate-intensity exercise at least twice per week for 30 to 60 minutes to enhance aerobic capacity and facilitate daily activities [52].

The effects of aquatic therapy were examined in a meta-analysis including 14 studies. Compared to non-aquatic exercises or no intervention, aquatic therapy demonstrated significant improvements in pain reduction, performance on the 6-minute walk test, total scores on the FIQ, and enhancements in vitality and general health as assessed by the Short Form Health Survey 36 (SF-36) [53].

The effects of aquatic therapy on sleep were evaluated in a review comprising 7 trials with 361 participants, of which 6 studies with 311 participants were included in a meta-analysis. The findings indicated that aquatic therapy enhances sleep quality. It is recommended that patients begin at a slightly lower intensity than their capacity allows, gradually increasing duration and intensity while engaging in aquatic therapy at low to moderate intensity at least twice per week [54].

A meta-analysis assessing the effects of aquatic therapy on sleep, which included 22 clinical trials, revealed considerable heterogeneity in the results. Although the Pittsburgh Sleep Quality Index did not show a statistically significant difference, an average reduction of 5.04 points in FIQ scores was observed, favoring aquatic therapy [55].

Another meta-analysis, involving 6 clinical trials, demonstrated that aquatic exercise for FM is superior to no intervention in reducing pain, fatigue, and the impact of the syndrome, as well as in alleviating depression and enhancing physical function and mental health in the short term. Furthermore, improvements were noted in syndrome impact, physical function, and mental health in the medium term. However, aquatic therapy did not show significant advantages over land-based exercises [56].

A Cochrane review with meta-analysis evaluated the effects of mixed physical exercise (aerobic, strength, and flexibility) on FM symptoms, analyzing 21 trials. The evidence was of moderate quality for primary outcomes and low for stiffness. The exercises improved physical function and led to a slight reduction in pain, with minimal reports of pain or fatigue during training and no adverse effects. Compared to control, mixed exercise likely improves quality of life, physical function, and fatigue; however, these effects may be small and clinically insignificant for some individuals. The evidence regarding long-term effects was classified as low quality [57].

### What are the benefits of strength training in the management of FM?

CONSENSUS: Strength and resistance exercises reduce pain and improve sleep and quality of life in patients with FM and therefore should be recommended.

Strength training has been evaluated in a systematic review of 22 studies, with interventions lasting between 3 and 21 weeks, performed twice weekly, and starting at intensities of 40% of one repetition maximum. The findings indicated reductions in pain and fatigue, as well as functional improvements [58].

Another systematic review examined strengthening programs lasting more than 2 weeks. Following Cochrane manual recommendations, 13 studies were included, with 9 analyzed in a meta-analysis. The results demonstrated improvements not only in pain and functionality but also in muscle strength and fatigue [59].

The impact of resistance training on sleep quality in FM patients was assessed in a systematic review of 6 studies, with 4 studies reporting significant sleep improvements. Resistance training was found to be superior to flexibility training and comparable to aerobic exercise. Interventions spanned 4 to 21 weeks, with sessions conducted 2 to 3 times per week at intensities ranging from 40% to 80% of one repetition maximum [60].

A systematic review analyzed the effects of strengthening exercises on mental health-related variables, including seven studies that reported data on depression and anxiety. Improvements in these parameters were observed, particularly with protocols that began at low intensity and gradually increased [61].

A 2024 systematic review of 15 randomized trials indicated that resistance training in women with FM significantly improves pain, functionality, and disease severity. However, the evidence was classified as low quality, highlighting the need for further studies. Strength training may also enhance treatment adherence, even in the absence of immediate symptom relief [62].

A 2024 meta-analysis of 11 clinical trials involving 530 FM patients demonstrated that resistance exercise, compared to no intervention, reduces pain, tender points, and depression while improving physical function. Compared to flexibility exercises, resistance training reduced the impact of FM, but its effects on pain control and physical function were similar to those of aerobic exercise [63].

### What are the therapeutic benefits of flexibility exercises in the management of FM?

CONSENSUS: Flexibility exercises in patients with FM demonstrate lower efficacy compared to aerobic and strength training exercises across most clinical parameters.

A systematic review incorporating four clinical trials comparing stretching exercises with aerobic training, strength training, or laser therapy demonstrated statistically significant improvements across nearly all evaluated parameters in both groups, with no single technique proving superior. However, the included studies exhibited low methodological quality and a lack of standardization in training protocols [64].

A systematic review of 12 randomized trials involving 743 participants indicated that flexibility training does not provide clinically significant improvements in quality of life, pain, fatigue, or physical function compared to aerobic exercise. Only a low certainty of improvement in stiffness was observed. The training was well tolerated, with a low dropout rate, but its long-term efficacy remains uncertain [65].

### Are there other modalities of physical activity/rehabilitation that are effective in the treatment of FM?

Consensus: Among alternative exercise modalities, vibrating platform training, exergaming, and Tai Chi have demonstrated evidence supporting their efficacy in the management of FM and may be considered as recommended therapeutic options.

Randomized studies suggest that vibrating platform training may enhance pain sensitivity, static balance, and quality of life in patients with fibromyalgia (FM), with improvements observed immediately following a 12-week intervention. However, these effects were not sustained after a three-month follow-up period [66, 67].

Two systematic reviews indicated that vibrating platform training can improve balance and reduce disability rates in FM patients. However, caution is warranted due to the limited number of trials and inconsistencies in study protocols [68]. Although short-term benefits have been reported, the long-term efficacy and feasibility of this intervention remain uncertain, necessitating further high-quality research [69].

A systematic review of nine randomized controlled trials, including 466 female patients, demonstrated that exergame training—a combination of video gaming and physical activity—has beneficial effects on general functioning, pain perception, quality of life, exercise capacity, health perception, and fatigue severity in FM patients. Exergame training is considered a promising non-pharmacological therapeutic option for FM management [70].

Tai Chi has demonstrated significant benefits for FM patients, including reductions in pain, fatigue, and depression, along with improvements in sleep quality and overall well-being, as evidenced by multiple studies [71–75]. It may be as effective as, or even more effective than, aerobic exercise, particularly when practiced over extended periods [74]. A meta-analysis of clinical trials supported these findings, highlighting the positive effects of Tai Chi in reducing FM symptoms and enhancing sleep quality [75]. Furthermore, clinical guidelines recognize Tai Chi as a viable therapeutic approach within the multidisciplinary management of FM, providing both physical and psychological benefits to patients [76].

### Is there evidence supporting the superiority of multimodal physical activity interventions over single-modality exercise in the management of FM?

CONSENSUS: The combination of aerobic and strength training exercises has been shown to be more effective than either modality alone.

Evidence indicates that the combination of different exercise modalities is more effective than isolated approaches in the management of FM. Two meta-analyses showed that aerobic exercise plus resistance provided greater improvements in quality of life, pain, and physical function compared with modalities performed alone. [77, 78]. Such findings suggest that a multimodal exercise approach offers a more comprehensive strategy for managing FM symptoms. [77] It is recommended that programs be 13 to 24 weeks long, with 30 to 60-minute sessions and progressive intensity. [78].

### Is acupuncture effective in the treatment of FM?

CONSENSUS: Acupuncture is recommended for the treatment of pain in FM.

Acupuncture, a traditional Chinese medicine practice encompassing both manual acupuncture and electroacupuncture, has demonstrated significant benefits in patients with fibromyalgia (FM), including improved quality of life, reduced depression, and pain relief when compared to placebo [79].

A meta-analysis investigating the effects of true acupuncture compared to a control group identified efficacy primarily in pain relief and enhancement of overall well-being [80].

Overall, acupuncture, including cranial acupuncture, has been shown to be effective in the short-term (up to six weeks) management of FM, particularly for pain reduction and decreased disability when combined with other treatments, such as pharmacological therapy [81, 82]. Additionally, acupuncture is considered a safe technique [83]. However, current evidence does not support its efficacy in reducing fatigue [82].

### Are neurostimulation techniques, such as transcranial direct current stimulation (tDCS) and repetitive transcranial magnetic stimulation (rTMS), effective in the management of FM?

CONSENSUS: tDCS and rTMS are effective in reducing pain associated with FM.

Neuromodulation, a therapeutic approach that stimulates the central nervous system (CNS) and/or peripheral nervous system, has been extensively studied for the management of FM. It has demonstrated efficacy in addressing various aspects of the syndrome, including pain intensity, pressure pain threshold, fatigue, catastrophizing, and quality of life [84–91].

The primary non-invasive neuromodulation techniques targeting the CNS tDCS and rTMS. The most commonly stimulated areas for pain relief are the dorsolateral prefrontal cortex (DLPFC) and the primary motor cortex (M1) [84].

A meta-analysis of 40 randomized controlled trials (RCTs) involving 1,541 participants found that high-frequency rTMS applied to M1 is effective in improving pain control and functionality in FM patients. tDCS applied to M1 was effective for pain, depression, and anxiety, with additional benefits for depression and functionality when applied to the left DLPFC [84]. In contrast, Sun et al. reported beneficial effects on pain in FM patients when low-frequency rTMS was applied to the DLPFC [86].

Another meta-analysis, which included 14 RCTs (6 tDCS and 8 rTMS) with 565 participants, found that tDCS applied to M1 was the only intervention that significantly reduced pain in the short and medium term. Both neuromodulation techniques demonstrated benefits, such as improvements in pressure pain threshold, catastrophizing, and quality of life when applied to M1, as well as reduced fatigue when applied to the left DLPFC [90]. However, their effects on anxiety and depression remain inconclusive [90, 92]. Nonetheless, a meta-analysis conducted by Yang CL et al. identified a moderate benefit of neuromodulation in alleviating depression in FM patients [93]. The effect size and neuroplastic changes induced by these interventions may be influenced by the duration of treatment [94].

### Is cognitive behavioral therapy effective treatment of FM? What about other forms of psychological therapy?

CONSENSUS: Cognitive Behavioral Therapy (CBT) and Acceptance and Commitment Therapy (ACT) have demonstrated evidence supporting their efficacy in the treatment of FM. However, there is currently no robust evidence supporting the effectiveness of other psychological therapy modalities.

Cognitive Behavioral Therapy (CBT) is a structured psychotherapeutic approach in which the therapist and patient collaborate to identify and modify maladaptive thoughts and behaviors that contribute to distress. The primary objective is to help patients reframe their perceptions of emotions and actions by analyzing significant life situations [95].

In Acceptance and Commitment Therapy (ACT), the focus is not on challenging the content of thoughts but rather on understanding their functional role within the patient’s life context. Instead of attempting to suppress or alter thoughts, ACT facilitates a change in the patient’s relationship with their thoughts and internal experiences [96].

Cognitive-behavioral therapies have demonstrated significant improvements in key symptoms of FM, including sleep quality, pain, anxiety, and depression. High-quality evidence from a systematic review and meta-analysis indicated that CBT is effective in reducing pain and enhancing quality of life in the short term [97].

CBT and ACT have been shown to be more effective than standard treatment in improving FIQ scores, pain, sleep, and depression, although they did not result in significant improvements in fatigue [98]. When combined with physical therapy interventions, CBT has contributed to reductions in depression and disability, in addition to enhancing the overall quality of life in FM patients [99].

Psychological interventions such as CBT and ACT have proven effective in alleviating symptoms of depression and anxiety in FM patients. As such, they can be reliably integrated into multidisciplinary treatment approaches, supporting emotional adaptation to chronic pain. Future research should prioritize the investigation of change mechanisms and multiple moderating factors to enable the development of personalized psychological interventions for FM management [100].

### Is there scientific evidence supporting the effectiveness of complementary and alternative therapies in the management of FM?

CONSENSUS: There is currently no scientific evidence supporting the efficacy of complementary and alternative therapies in the treatment of FM.

Alternative and complementary therapies have gained increasing attention in the management of FM; however, their efficacy remains uncertain due to methodological limitations and conflicting study results.

Traditional Chinese medicine, when combined with conventional therapy, has demonstrated greater efficacy in pain relief and overall health improvement. However, these findings should be interpreted with caution due to the heterogeneity of studies and potential adverse events [101].

Hyperbaric oxygen therapy has shown benefits in alleviating pain, fatigue, and sleep disturbances, but the lack of robust systematic reviews prevents definitive conclusions regarding its efficacy and safety [102].

Myofascial release and lymphatic drainage therapies provide moderate evidence for pain reduction and improvements in sleep quality and overall quality of life, although available data remain limited [103, 104].

Among manual therapies, only general osteopathic treatment has demonstrated positive effects on pain, whereas other modalities such as cupping, spinal manipulation, homeopathy, and topical capsaicin lack sufficient or conclusive evidence to support their recommendation [105–107].

Balneotherapy and hydrotherapy require further investigation to establish their long-term benefits [108].

Conversely, massage therapy administered for a duration of five weeks or more has shown immediate benefits in reducing pain, anxiety, and depression, making it a viable therapeutic option. However, high-quality evidence supporting its formal recommendation remains lacking [109].

Further well-designed studies are necessary to establish the role of these complementary and alternative approaches in the management of FM.

### What is the role of spirituality and religiosity in the management o FM?

Consensus: Spirituality and religiosity play a significant role in the management of FM.

Spirituality and religiosity may play a significant role in the management of fibromyalgia (FM). For many individuals, these practices provide emotional and psychological support, aiding in coping with chronic pain and the challenges associated with the condition. Studies suggest that spiritual and religious practices can help reduce stress, anxiety, and depression—common comorbidities in FM—while fostering a sense of peace, purpose, and resilience, thereby facilitating adaptation to physical symptoms.

Activities such as meditation and prayer, commonly integrated into spiritual practices, have also been associated with improved sleep quality and pain relief. However, it is essential to recognize that spirituality and religiosity serve as complementary approaches rather than substitutes for conventional medical treatment, which includes pharmacotherapy, physical therapy, structured exercise programs, and psychological support [110, 111].

## Conclusion

The management of fibromyalgia (FM) requires a comprehensive, interdisciplinary approach integrating pharmacological and non-pharmacological strategies. This consensus highlights the importance of patient monitoring, education, and multimodal interventions, including exercise, psychological therapies, and neuromodulation techniques. While certain therapies, such as aerobic and resistance training, cognitive-behavioral therapy, and acupuncture, demonstrate efficacy, others require further high-quality research to establish their clinical relevance. These updated guidelines reinforce evidence-based recommendations, aiming to optimize patient outcomes and improve quality of life through personalized and integrative care.

## Data Availability

No datasets were generated or analysed during the current study.

## Abbreviations

AAPT: ACTTION-American Pain Society Pain Taxonomy
ACR: American College of Rheumatology
ACT: Acceptance and Commitment Therapy
CAM: Complementary and Alternative Medicine
CBT: Cognitive Behavioral Therapy
CNS: Central Nervous System
CS: Central Sensitization
CSI: Central Sensitization Inventory
DLPFC: Dorsolateral Prefrontal Cortex
FIQ: Fibromyalgia Impact Questionnaire
FIQR: Revised Fibromyalgia Impact Questionnaire
FM: Fibromyalgia
FODMAP: Fermentable Oligosaccharides, Disaccharides, Monosaccharides, and Polyols
FSQ: Fibromyalgia Survey Questionnaire
HRQoL: Health-Related Quality of Life
M1: Primary Motor Cortex
OA: Osteoarthritis
OMERACT: Outcome Measures in Rheumatology
PNS: Peripheral Nervous System
PROs: Patient-Reported Outcomes
QoL: Quality of Life
RA: Rheumatoid Arthritis
RCT: Randomized Controlled Trial
rTMS: Repetitive Transcranial Magnetic Stimulation
SBR: Brazilian Society of Rheumatology
SF-36: Short Form Health Survey 36
SpA: Spondyloarthritis
SSS: Somatic Symptom Severity Score
tDCS: Transcranial Direct Current Stimulation
WPI: Widespread Pain Index
